# Outcome of upper respiratory tract infections in healthy children: Antibiotic stewardship in treatment of acute upper respiratory tract infections

**DOI:** 10.12669/pjms.36.4.1420

**Published:** 2020

**Authors:** Ejaz Ahmed Khan, Mazhar Hussain Raja, Shehla Chaudhry, Tehreem Zahra, Salman Naeem, Masuma Anwar

**Affiliations:** 1 Ejaz Ahmed Khan, MBBS, MD. Department of Pediatrics, Shifa International Hospital Ltd, Shifa Tameer-e-Millat University, Islamabad, Pakistan; 2 Mazhar Hussain Raja, MRCP, MRCPC. Department of Pediatrics, Shifa International Hospital Ltd, Shifa Tameer-e-Millat University, Islamabad, Pakistan; 3 Shehla Chaudhry, FCPS. Department of Pediatrics, Shifa International Hospital Ltd, Shifa Tameer-e-Millat University, Islamabad, Pakistan; 4 Tehreem Zahra, MBBS. Department of Pediatrics, Shifa International Hospital Ltd, Shifa Tameer-e-Millat University, Islamabad, Pakistan; 5 Salman Naeem, MBBS. Department of Emergency Medicine, Shifa International Hospital Ltd, Shifa Tameer-e-Millat University, Islamabad, Pakistan; 6 Masuma Anwar, MBBS. Department of Pediatrics, Shifa International Hospital Ltd, Shifa Tameer-e-Millat University, Islamabad, Pakistan

**Keywords:** Upper respiratory tract infections, Children, Antibiotics, Antibiotic stewardship

## Abstract

**Objective:**

The objective of the study was to assess the outcome of upper respiratory tract infections (URTI) in healthy children.

**Methods:**

This descriptive study was conducted on 314 children aged 3-36 months in the paediatric outpatient clinic and emergency department with symptoms of URTI (fever, cough, rhinorrhoea) for ≤5 days. Patient’s demographics, clinical features, laboratory data and outcome were recorded. Follow up phone calls were made to parents on day 7 (response 93.6%) and day 14 (response 94.6%) to record outcome.

**Results:**

A total of 314 children with URTIs were included. Majority (57.6%) were males and <1year of age (40%). Common manifestations of URTI were fever (89%), cough (79%), rhinorrhoea (62%), pharyngitis (79%) and conjunctivitis (46%). More than half (53%) had history of contact with URTI in a family member. Mean duration of symptoms was 2.7±1.3 days. Majority (93%) of children were given supportive treatment and only 6.7% received antibiotics initially. Most of children (76%) recovered within one week and 91.8% within two weeks with supportive care only. Only 4% children were hospitalized and 12% required follow up visit of which 16% needed oral antibiotics. Complications or deaths did not occur.

**Conclusions:**

Majority of URTIs in healthy children resolved with supportive treatment and do not require antibiotics. Antibiotic stewardship in simple URTIs should be practiced using awareness and advocacy campaigns.

## INTRODUCTION

Upper respiratory tract infection (URTI) is one of the most common childhood illnesses with mostly an acute, self-limited course.^1^,^2^ It is caused by a variety of infectious agents but mostly respiratory viruses (61%).^2^ *Rhinoviruses*, *respiratory syncytial virus* (*RSV*), *influenza viruses*, *parainfluenza viruses* and *adenoviruses* are commonly responsible for URTIs in preschool children accounting for at least 50% of colds in both children and adults with admission in 180 per 10,000 children.^2^

Diagnosis of URTI is made on clinical grounds. Laboratory tests are usually not needed in healthy children with uncomplicated URTI. However, rapid viral testing (direct immunofluorescent-antibody staining) and reverse transcriptase-PCR may be done for rapid diagnosis in emergency rooms and sometimes in outpatient clinics. Most of these children recover completely. Antibiotics are indicated for specific diagnosis such otitis media or sinusitis if associated or follow URTI.

The use of antibiotics in URTI is very common despite the self-limiting nature of most viral infections.^3^ Antibiotic stewardship principal dictates that antibiotics should not be used in these viral infections in children and adults.^4^,^5^ Antibiotic stewardship is a “collective set of strategies to improve the appropriateness and minimize the adverse effects of antibiotic use, decrease resistance, toxicity and costs and to promote the selection of the optimal antibiotic regimen, dose, duration and route of administration”. In Pakistan URTI epidemiology in children has not been well documented. Only few studies exist related to influenza, respiratory viruses, severe pneumonia and bronchiolitis.^6^-^8^ Unfortunately up to 70% of patients with URTI antibiotics are prescribed.^9^ This clinical study aims to assess the outcome of URTIs in healthy children and to discourage the inappropriate use of antibiotics.

## METHODS

This is a descriptive study conducted at Department of Pediatrics, Shifa International Hospital from January 2014 to February 2015 after approval from Institutional Review Board and Ethics Committee (Ref: IRB# 329-178-2014 dated January 23, 2014). All healthy children (age 3-36 months) presenting in Pediatrics outpatient clinic and emergency department with symptoms of URTI (including fever, cough, rhinorrhea) for ≤5 days were enrolled in the study after written informed consent from the parents. Patients were given treatment as per standards and at the discretion of their respective paediatricians, which included supportive treatment such as antipyretics, nebulization, saline nasal irrigation and to increase fluids. Counselling was given for the self-limiting course of URTI, specific indications for antibiotic use and to return if there is deterioration. Hospitalization, antibiotics or investigations were done in selected cases only. Follow-up phone calls were made to parents on day 7 (response 93.6%) and day 14 (response 94.6%) to record outcome. Outcome variables included % of children given or discontinuing antibiotics, % requiring hospitalization or laboratory evaluation, % children with resolution of symptoms on day 7 and 14.

The sampling technique was purposive and consecutive. Sample size of 314 (335±10%) was calculated by taking 0.05 at 95% confidence level, 0.05 absolute precision, prevalence^3^ of 68% by using WHO sample size calculator.

Patients’ demographic details, underlying illnesses, immunization history, and clinical features on examination, laboratory data and outcome were recorded. Demographics, clinical, features, management and outcome were presented as mean ± standard deviation (SD) for quantitative variables or percentage for qualitative variables.

## RESULTS

A total of 314 children with URTI were enrolled for the study. Majority were males, infants (<1year of age), had a history of contact with family member with URTI with common signs and symptoms of URTI (Table-I). On initial visit, majority (93.3%) of children were given supportive treatment and only 6.7% required an antibiotic based on specific focus of infection (Table-II). Antibiotics were discontinued at presentation in 18.5% children with most commonly prescribed cefixime (41%). Specific diagnoses were present in 17.5% children with otitis media in 31% that required an antibiotic and amoxicillin was the commonly prescribed antibiotic. A total of 11.8% children required a follow-up clinic visit with 78% having same symptoms and only 16% (6/37) required an antibiotic but of these 83% had recovered at the end of second week.

**Table-I:** Demographics and clinical characteristics of children with URTI in 314 children.

Parameters	N (%), Mean, Range
Total	314 (100)
*Demographics*	
• Males	181 (57.6)
• Age	
• <1 years	124 (39.5)
• Mean age in months	16.6±9.5 (3-36)
*History*	
• Contact in the family with viral URTI	168 (53.5)
• Fully Immunized	309 (98.4)
*Mode of visit*	
• Outpatient clinic	294 (93.6)
• Emergency Department	20 (6.4)
Mean documented temperature	101.4±1.2ºF (99-104)
Mean duration of symptoms	2.7±1.3 days (1-5)
*Presenting Symptoms*	
• Fever	279 (88.9)
• Cough	248 (79)
• Rhinorrhea	194 (61.8)
• Anorexia / reluctance feed	112 (35.7)
• Vomiting	77 (24.5)
• Disturbed sleep	23 (7.3)
• Ear ache	8 (2.5)
*Physical Signs*	
• Erythema of pharynx	249 (79.3)
• Conjunctivitis	146 (46.5)
• Rash	17 (5.4)
• Rhonchi/crepitations	3 (1%)
*Associated Clinical diagnosis*	
• Otitis media	17 (5.4)
• Croup	12 (3.8)
• Bronchiolitis	10 (3.2)
• Febrile seizures	7 (2.2)
• Measles	2 (0.6)

URTI: upper respiratory tract infections

**Table-II:** Management and outcome of children with URTI.

	N (%)
Total Children with URTI	314 (100)
Supportive therapy	293 (93.3)
Hospitalization	12 (3.8)
• On initial visit	12 (3.8)
• Follow up visit	0 (0)
*Indications*	
• Worsening or persisting tachypnea	8 (2.5)
• Oxygen therapy	2 (0.6)
• Parental anxiety	1 (0.3)
• Mechanical ventilation	1 (0.3)
Total Investigations	9 (3)
• CBC	8 (2.5)
• CXR	5 (1.6)
• Nasopharyngeal for rapid viral antigen	1 (0.3)
• Blood culture	0 (0)
• Ear swab Culture	0 (0)
*Antibiotic use*	
• Stopped at initial visit	58 (18.5)
• Given at initial visit	21 (6.7)
• At follow up visit	6 (1.9)
• Overall Antibiotics prescribed	27 (8.6)
*Outcome on Day 7*	
• No response	24 (7.6)
• Worse or persistence of symptoms	70 (24)
• Recovered completely	220 (76)
*Outcome on Day 14*	
• No response	21 (6.7)
• Worse or same	24 (8.2)
• Recovered completely	269 (91.8)
Follow-up clinic visits	37 (11.8)

URTI: upper respiratory tract infections, CBC: complete blood count, CXR: chest x-ray, OM: Otitis media

* Antibiotics given on initial presentation included amoxicillin in 17 (14 for OM) and amoxicillin-clavulanate in 2 children (both OM).

** Antibiotics given on follow-up included amoxicillin in 3 (for OM) and amoxicillin-clavulanate in 1 child.

Only 3.8% children were hospitalized for persistent or worsening symptoms, unable to give treatment at home or due to parental anxiety (Table-II). Management included nebulization (saline with or without an inhaled bronchodilator), intravenous fluids, oxygen and mechanical ventilation. Empiric antibiotics were given to 8 children (75%) and discontinued in <72 hours in 5 (62.5%) of these children. The duration of hospitalization was <2 days in majority (8/12, 75%) of these children. Follow-up phone calls were not answered in 7.6% and 6.7% at first and second week respectively. At end of week one, 76% children had recovered completely; 24% had same or worsening symptoms. At the end of week two 91.8% children had recovered completely and only 8.2% had same or worsening symptoms. Overall only 8.6% children required an antibiotic for a specific diagnosis. Complications or death did not occur.

## DISCUSSION

Our study found that in 92% of healthy children simple URTI resolved with supportive treatment only by second week of illness. Antibiotic were required in only approximately 9% of children with otitis media as being the commonest indication. Acute URTI in children are common with average of 8-10 episodes per year/child that resolve in majority without any specific therapy.^1^-^3^ Surveillance studies from community, emergency visits and hospitalizations show the importance of various viral pathogens such as *rhinoviruses, RSV, influenza viruses, parainfluenza viruses*, and *adenoviruses*.

In Pakistan the exact etiology of URTIs has not been described. However a country-wide lab-based surveillance system (children and adults) for influenza-like-illness and Severe Acute Respiratory Illness across Pakistan showed that *influenza virus* was detected in 24% samples (72% *Influenza* type A and 28% *influenza* type B viruses).^6^ A rural community-based prospective cohort active surveillance for respiratory viruses in infants <2 years old from Sindh showed different viral pathogens in 77.8% nasopharyngeal (*enterovirus* and *rhinovirus* (51.7% infants), *parainfluenza virus* type III (8.3%), and *RSV* (5.7%).^7^ Another surveillance study of bronchiolitis and pneumonia in hospitalized <2 years old showed different viral pathogens in 75% cases (*RSV* 67%: *Influenza A*: 24.5%, *Influenza B*: 7%, *Adenovirus*: 8.4% and *human metapneumovirus*: 5.2%).^8^

Clinically infants and children with URTI will present with a set of signs and symptoms lasting 7-14 days.^1^,^2^ Most of these children present with fever, cough, vomiting, rhinorrhoea and sore throat. Physical signs are non-specific, but may include clear to cloudy nasal discharge, conjunctivitis, oral ulcers and pharyngeal erythema. URTI has a higher prevalence during the fall and winter months as different viruses move through the community in a predictable manner such as the common cold.^1^ Many of these viruses may also cause other characteristic syndromes in children such as influenza like illness (*influenza* A and B), bronchiolitis (*RSV)*, croup (*Parainfluenza viruses)*, herpangina (*coxsackie A viruses*) and pharyngoconjunctival fever (*Adenoviruses*) and some may require hospitalization.

Treatment of URTI is generally symptomatic with antipyretics, saline nasal irrigation, adequate hydration and a humidifier. Antihistamines, decongestants, antitussives, and expectorants, have not demonstrated any benefit.^5^ There is no justification for antibiotics in the treatment of URTI such as the common cold.^3^,^5^ American Academy of Pediatrics on Infectious Diseases has outlined some basic principles of antibiotic prescribing for URTIs in pediatrics including determining the likelihood of bacterial aetiology, weighing the benefits versus harm of antibiotics and implementing judicious antibiotic prescribing.^4^ However, million of antibiotic courses are dispensed in outpatient setting with at least 30% of antibiotics prescribed being unnecessary.^3^,^5^

In Pakistan physicians including paediatricians continue to use antibiotics excessively in most URTIs. Pakistan has one of the highest (62-70% of patients) prescription rate for antibiotics.^10^-^12^ This overuse and abuse was more common among general practitioners, public hospitals for costly antibiotics and 3^rd^ generation cephalosporins.^12^,^13^ Availability of over the counter antibiotics and self-medication with mostly a broad-spectrum antibiotic for fever and sore throat is common in Pakistan.^13^ These broad-spectrum antibiotics are one of the main drivers of antibiotic resistance. In our study antibiotics given included broad-spectrum cefixime in 41% of children that were discontinued and given only supportive care. A questionnaire based study from Peshawar about use of antibiotics in children for common URTI showed that common practices seen were physician prescribed antibiotics (58%), family recommendation (27%), usefulness in fever or viral infections (35-25%) and self-medication (25%).^14^

The futility of antibiotics for self-limiting viral URTIs has been well documented. Guidelines for management of URTIs in children advocate selective use of antibiotics for conditions such as otitis media and sinusitis only.^15^,^16^ A non-Cochrane meta-analysis of 80 studies and a Cochrane systematic review of 10 high-quality randomized control trials showed only small benefit for acute otitis media in children.^16^ A Cochrane review of 27 trials on antibiotic use for sore throat also showed limited role for antibiotics.^17^ Antibiotic stewardship is a strategy to reduce the irrational antibiotic use in both inpatient and outpatient settings.^18^ It specifically can be used to control antibiotic use in URTIs such as common cold and pharyngitis.^4^,^5^,^19^ Other evidence about specific stewardship interventions that are well documented include communication skills, training and education.^20^ One proven antibiotic stewardship strategy is delayed prescription.^21^ A delayed antibiotics strategy may significantly reduce unnecessary antibiotic use for respiratory infections such as otitis media.^4^

Antibiotics do not prevent secondary bacterial infection, may cause significant side effects and contribute to increasing antimicrobial resistance and costs.^22^ Awareness, education and training through campaigns about antibiotic stewardship must be done for all physicians at national level. A number of online antibiotic stewardship courses, including free courses, are available that may be used by clinicians and other healthcare workers to update and gain antibiotic stewardship skills.^23^,^24^ Vaccination and good infection control is also advocated for preventing transmission of the common viral illnesses. Simple practice of frequent hand washing, following cough etiquettes will lead to fewer illnesses and less need of antibiotics. Yearly administration of the influenza vaccine to prevent influenza infection and its complications will also help in curtailing the use of unnecessary antibiotics.^25^

## CONCLUSIONS

Our study demonstrated that most healthy children with URTIs do not need any antibiotics and less need for hospitalization, investigations or follow-up visits without any adverse outcome. Antibiotic stewardship in simple viral URTIs should be practiced using educational awareness and advocacy campaigns for all practitioners at national level.

### Author’s Contribution

**EAK:** Contributed to the initial conception and design of the manuscript.

**EAK, MHR, SC, TZ, SN & MA:** Did the acquisition and assembly of the clinical data and were responsible for revising the manuscript critically for important intellectual content.

**EAK, MHR & SC:** Contributed to the critical revision.

**EAK, MHR & SC:** Were responsible for designing, analyzing data, and revising the manuscript critically for important intellectual content.

All authors contributed to writing and approval of the final version of the paper.
